# Buckling Analysis of a Large Shelter with Composites

**DOI:** 10.3390/ma14237196

**Published:** 2021-11-25

**Authors:** Sheldon Wang, Jianyao Mou

**Affiliations:** 1 McCoy School of Engineering, Midwestern State University, 3410 Taft Blvd., Wichita Falls, TX 76310, USA; 2 CRYOPAK/TCP RELIABLE, 551 Raritan Center Parkway, Edison, NJ 08837, USA; jmou@cryopak.com

**Keywords:** finite element analysis, collapse, nonlinear analysis, buckling, deployable shelter

## Abstract

We present here linear and nonlinear finite element analyses of a newly designed deployable rapid assembly shelter (DRASH J) manufactured by DHS Systems. The structural analysis is carried out in three stages. Firstly, single composite tubes (struts) under three-point bending are modeled with five layers of orthotropic materials in three different orientations and the simulation results are compared with the actual test data for validation. Secondly, a comprehensive structural model for the entire shelter is constructed with the consideration of two types of strut scissor points, namely natural and forced scissor (crossing) points, as well as partial-fixed hub joints, which allow rotations along individual hub slots (grooves). Finally, a simplified structural model is created by introducing fixed joints for the scissor points as well as rigid links for the hubs. With sufficient verifications with experiments and different modeling methods, linear and nonlinear finite element analyses are then carried out for both the comprehensive and simplified shelter models. Based on the simulation results, we are able to identify a few critical issues pertaining to proper design and modifications of such shelter systems, such as various end wall supports pertaining to the overall structural stability.

## 1. Introduction

**Figure 1:**
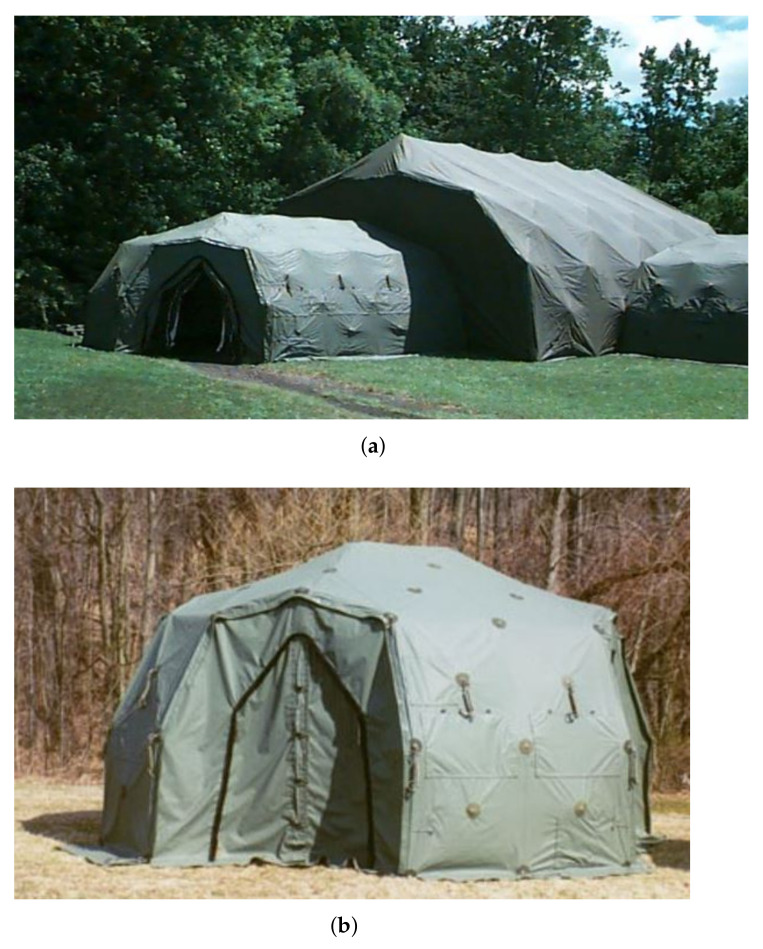
Typical DRASH shelters. (**a**) JS. (**b**) XBS.

The deployment of deployable rapid assembly shelters (DRASH) in a field emergency can be accomplished minutes after arrival at the crisis areas for both military and civilian missions. DHS Systems provides state-of-the-art technology in such portable sheltering. Figure 1 depicts two typical DRASH Shelters which can be easily handled by six people within 20 to 30 min using a low pressure air bladder. The J shelter features the same proven DRASH technology as the smaller XB shelter and is currently one of the largest single unit shelters manufactured by DHS Systems. The J model considered in this analysis measures externally 33.66L×34.16W×17.97H ft and provides more than 1100 ${ft}^{2}$ of usable interior floor space.

The key frame of the shelter comprises of composite tubing structures with pre-attached interior and exterior covers. So far, limited study has been performed for the entire structure stability of the J shelter with respect to snow load. The research presented in this work is particularly important because it provides the first linear and nonlinear structural analysis for the entire J shelter as well as necessary verification comparisons with both experiments and different modeling methods.

The structural analysis is carried out with three levels of modeling. Firstly, single composite tubes (struts) under three-point bending are modeled with five layers of orthotropic materials in three different orientations and the simulation results are compared with the test data for validation. Secondly, a comprehensive structural model for the entire shelter is created with two different types of scissor (crossing) points, namely natural and forced scissor points, as well as a realistic hub model which allows one rotational degree of freedom within the hub slot (groove). Finally, a simplified structure is introduced, in which all scissor points are replaced with fixed joints, whereas hubs are substituted by a set of translational rigid links. For the entire structure analysis, all struts are modeled as isotropic and homogeneous three-dimensional beam elements of which the effective Young’s modulus and the bending rigidity are derived in the first stage based on both simulation and experimental data. In the following section, we illustrate the mathematical model and the key physical assumptions corresponding to the initial assemblage process, the mechanical behavior of the composite beams, and various strut connections within the shelter structure. The key findings and assumptions will be reiterated in Section 3 as well as the concluding section.

## 2. Mathematical Models

In general, each strut consists of five layers of fiber-reinforced composite materials wrapped around in three different orientations to produce high strength with minimum weight. Since the J shelter is composed of over a hundred struts interconnected with pins and hubs to form the main shelter arch, it could be an insurmountable task to model the entire shelter structure with such composite shell models. Thus, for simplicity, the composite struts are modeled as three-dimensional isotropic and homogeneous beam elements in the global models. The effective Young’s modulus is derived from the three-point bending test and can be expressed as

(1) $$E=\frac{4Pl^{3}}{3\delta \pi (d_{o}^{4}-d_{i}^{4})},$$

where $d_{o}$ and $d_{i}$ stand for the outer and inner tube diameters, *P* and δ represent the maximum load and deflection of the three-point bending test, and *l* is the span of the beam.

**Figure 2:**
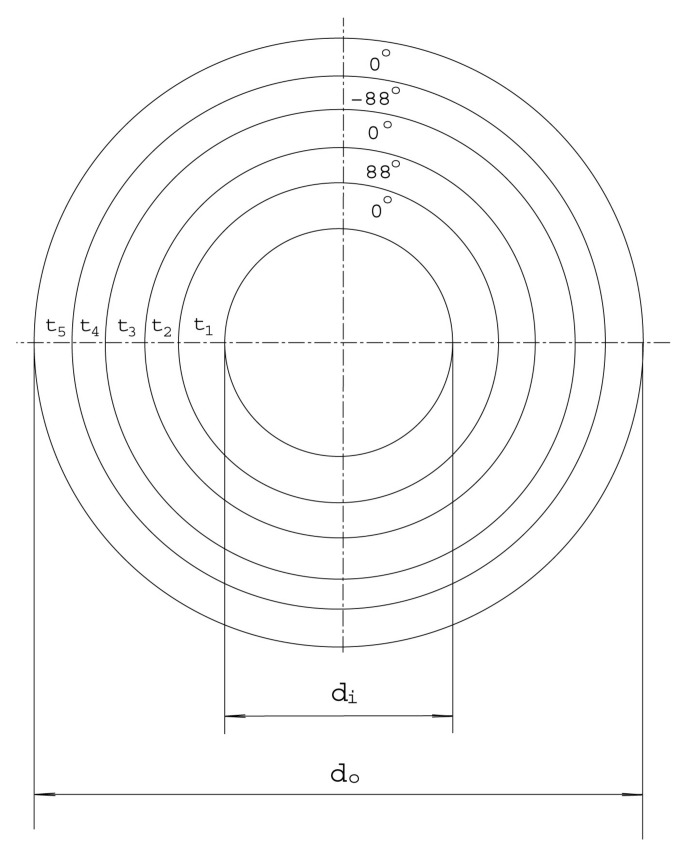
A typical cross-section of a strut.

In Figure 2 we show a typical cross section of a composite strut. For orthotropic materials, we normally have three pairs of independent Young’s and shear moduli in the corresponding principal directions, which are often aligned with the matrix orientations. In this study, we choose, according to references [1,2], $E_{m}=6.30\;Msi$, $E_{h}=1.53\;Msi$, $E_{z}=1.91\;Msi$, $G_{mh}=0.0817\;Msi$, $G_{hz}=0.659\;Msi$, and $G_{zm}=0.648\;Msi$, where the subscripts *m*, *h*, and *z* represent the meridional (axial), hoop (circumferential), and radial directions of the composite tube, respectively. The orientation of an orthotropic layer refers to the angle between the meridional direction and the major orthotropic material principal direction. Of course, Poisson’s ratios must satisfy the usual compatibility condition $\frac{E_{a}}{\nu_{ba}}=\frac{E_{b}}{\nu_{ab}}$ for orthotropic materials [3]. In this work, we choose $\nu_{mh}=0.075$, $\nu_{hz}=0.328$, and $\nu_{zm}=0.278$. The other associated orthotropic properties, according to Refs. [1,2], are listed in Table 1.

For the structural analysis, we start with linear analyses of both comprehensive and simplified models. Then, we employ the linearized buckling, collapse, and nonlinear analyses, which take into account the geometrical nonlinearities, in order to calculate the maximum snow load that the entire shelter structure can sustain prior to structural instability. Furthermore, the reaction forces at the shelter supports are also calculated with respect to the collapse load.

The basic concept of nonlinear structural analyses can be best illustrated in the following iteration procedure

(2) $${}^{t+\Delta t}K^{\left(i\right)}\Delta U^{\left(i\right)}{=}^{t+\Delta t}R{-}^{t+\Delta t}F^{\left(i\right)},$$

where K, U, R, and F are the tangent stiffness matrix, the nodal displacement unknown vector, the external load vector, and the equivalent internal nodal force vector corresponding to the element stresses, respectively, and the incremental iteration at the time step t+Δt starts from:

$${}^{t+\Delta t}U^{\left(0\right)}={}^{t}U,{}^{t+\Delta t}U^{\left(i+1\right)}={}^{t+\Delta t}U^{\left(i\right)}+\Delta U^{\left(i\right)},{\mathrm{and}\;}^{t+\Delta t}F^{\left(0\right)}{=}^{t}R.$$

It is obvious that in the linear analysis, the tangent stiffness matrix K is equivalent to the constant structural stiffness matrix. Notice that, in this work, we consider the quasi-static analysis, which means that the time step corresponds only to the loading sequence and no mass matrix or inertia is involved. Naturally, collapse or buckling refers to the load and displacement conditions per se, at which the tangent stiffness matrix is singular. In the linearized buckling analysis, suppose we have two tangent stiffness matrices at *t* and t−Δt, to find a particular external load ${}^{\tau }R$ such that

(3) $$det{(}^{\tau }K)=0,$$

we can solve the following eigenvalue problem, using the two equations for a load scaling factor λ, as listed below:

(4) $$\begin{array}{ccc} {}^{\tau }K & = & {}^{t-\Delta t}K+\lambda {(}^{t}K{-}^{t-\Delta t}K),\\ {}^{\tau }R & = & {}^{t-\Delta t}R+\lambda {(}^{t}R{-}^{t-\Delta t}R). \end{array}$$

By substituting the first equation in Equation (4) into Equation (3), we obtain the eigenvalue problem with respect to the linearized buckling analysis,

(5) $${}^{t-\Delta t}K\phi =\lambda {(}^{t-\Delta t}K{-}^{t}K)\phi ,$$

in which the eigen vector, or the buckling mode shape ϕ, corresponds to the eigenvalue, or the buckling scaling factor λ.

Consequently, implementing the second equation in Equation (4), we obtain the corresponding buckling load ${}^{\tau }R$.

In fact, Equation (5) can also be rewritten as

$${}^{t}K\phi =\xi^{t-\Delta t}K\phi ,$$

with

$$\xi =\frac{\lambda -1}{\lambda }.$$

To obtain the collapse point as well as the load-displacement curve beyond the initial buckling point, i.e., to derive the post-buckling curve, we can use a displacement-controlled (LC) or load-displacement-constraint method (LDC) loading sequence. The basic concept of the LDC method is to derive the load scaling factor ${}^{t+\Delta t}\lambda$, which corresponds to buckling modes and satisfies

(6) $${}^{t+\Delta t}\lambda R-{}^{t+\Delta t}F=0,$$

by employing the similar Newton–Raphson iteration as illustrated in Equation (2),

(7) $${}^{t+\Delta t}K^{\left(i\right)}\Delta U^{\left(i\right)}={}^{t+\Delta t}\lambda^{\left(i\right)}R-{}^{t+\Delta t}F^{\left(i\right)},$$

with ${}^{t+\Delta t}\lambda^{\left(i\right)}={}^{t+\Delta t}\lambda^{\left(i-1\right)}+\Delta \lambda^{\left(i\right)}$.

Since we introduce an additional unknown, λ, we need to have an additional equation, for example, a constraint, which governs the so-called spherical constant arc length Δl,

(8) $${\left(\lambda^{\left(i\right)}\right)}^{2}+\frac{{U^{\left(i\right)}}^{T}U^{\left(i\right)}}{\beta }={\left(\Delta l\right)}^{2},$$

with $U^{\left(i\right)}{=}^{t+\Delta t}U^{\left(i\right)}{-}^{t}U$, where β is a normalization factor. A detailed discussion of collapse analysis is available in references [4,5,6,7,8].

## 3. Finite Element Modeling

The computer simulations presented in this study are performed with the ADINA program, which consists of the program on heat transfer in solids ADINA-T, the program on displacements and stresses ADINA, the program on fluid flows and heat transfer ADINA-F, the pre-processor ADINA-IN, and the post-processor ADINA-PLOT. Both the geometry and the corresponding mesh can be generated in ADINA-IN. A detailed description of the recent development of the ADINA program is available in references [9,10,11].

In this section, we discuss some in-depth modeling procedures, namely, the three-point bending of a single composite strut, the natural and forced scissor points of two crossing struts, and the hubs.

### 3.1. Three-Point Bending

**Figure 3:**
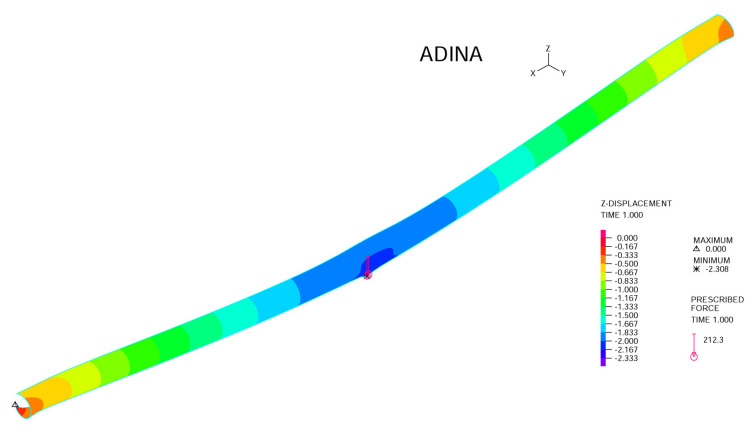
The three-point bending of a composite tube with P=424.5lbf.

As shown in Figure 3, we simulate a single strut simply supported at its two ends with a maximum center point load (*P*). The cross-sectional properties of the composite tubes are listed in Table 1. The inner and outer diameters ($d_{i}$ and $d_{o}$), the span of the three-point bending (*l*), and the center point loading (*P*) are 0.874 and 1.0654in, 28.5in, and 424.5lbf, respectively.

The three-point bending experiment of the J shelter has produced a deflection of 2.26in, which is very close to the ADINA simulation result, namely 2.31in, with comparable boundary conditions. It is clearly shown that this type of modeling with multi-layer composite shell model is a valid approach. However, to be safe and rigorous and, more importantly, to be able to handle many such multi-layer composites in a complex structure, we need to employ the effective Young’s modulus from the simulation data based on Equation (1).

### 3.2. Strut Scissor Points and Hubs

**Figure 4:**
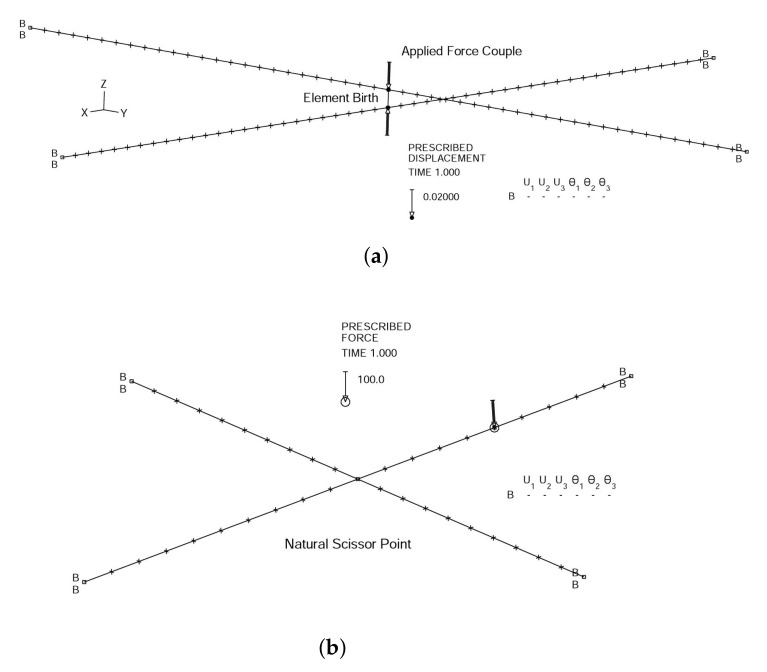
Different modeling of scissor points, namely with (**a**) an element birth and (**b**) with a natural scissor point.

In the J shelter, there are numerous scissor points between struts which can be divided into two basic groups. One of them is the so-called natural scissor point group which does not introduce significant initial stress within the composite strut, and the other one is called the forced scissor point group, which does introduce significant initial bending, i.e., initial stress, during the structural assemblage. Notice, however, although the struts in such scissor points are connected with a pin, if we model the composite tube as a beam, the rotational degrees of freedom along the struts are continuous just as all the translational degrees of freedom. To verify our assumptions, we introduce two sets of mathematical models as shown in Figure 4. The initial bending is accomplished by assigning a specially chosen external force couple, as depicted in Figure 4, to connect the two struts together at the intersection point and then release (or create) a new finite element (very stiff with respect to the struts) to represent the fixed pin connection. With regard to the natural scissor point, as shown in Figure 4, at the geometrical intersection point, we can assign four different points occupying the same geometrical space and test the following five separate scenarios:

Case 1 Four lines share a common node at the intersection point, which represents a completely fixed joint.
Case 2 Four lines rigidly linked through four end points occupying the same geometrical point, which represents the same physical model as Case 1, however, its finite element procedure is different.
Case 3 Four lines share a common point, at which only the translational degrees of freedom are constrained.
Case 4 Rigid links are separately employed along the two crossing struts, which means, within the crossing struts, only the translation degrees of freedom are constrained.
Case 5 A truss element is introduced to physically connect two points at the vicinity of the intersection point.

The center point deflection results are used as measures of the accuracy of various constraints and boundary conditions. It is obvious from Table 2 that other than the fully pinned connection (Case 3), for the natural scissor point, we can practically choose any one of the other four constraints. In this study, instead of choosing Case 4, which is clearly the closest to the physical connection within the shelter, for simplicity, we select Case 5. Therefore, the element birth and death capabilities of ADINA allow us to introduce initial bending into the forced scissor point group of the comprehensive model.

**Figure 5:**
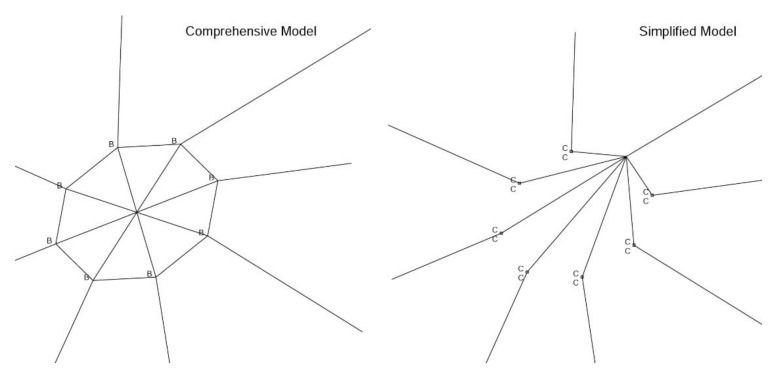
Two different hub constraints, namely comprehensive and simplified models.

With respect to the hubs, as illustrated in Figure 5, for the comprehensive and simplified structural models, we consider two possibilities of boundary conditions, namely a fully-fixed hub and a partial-fixed hub, which reserves one rotation degree of freedom to account for the rotation within the hub slot, just as the actual hub. Notice, however, reflected from simulation results, both hub models actually produce similar solutions.

### 3.3. Global Models

**Figure 6:**
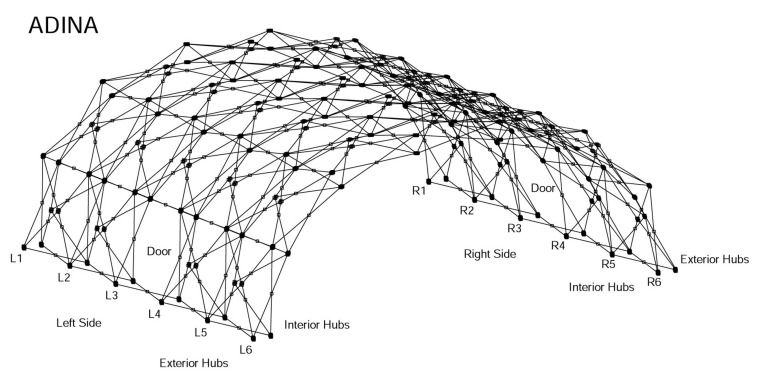
The entire structural model of the J shelter without risers.

The entire structure models, as shown in Figure 6, are based on the geometrical locations of beam joints created in AutoCAD. Similar finite element analyses of these types of complex structures have been reported in references. [12,13]. In this study, we focus on both the linear elastic response and linearized buckling analysis of the entire structures as well as the nonlinear analyses. In the finite element modeling, the implementation of the comprehensive model is a very difficult task, considering the large number of scissor points and hubs, and in particular, the groove skew systems with respect to the individual rotational degrees of freedom. Therefore, it is necessary to simplify the entire structural model. To be more specific, for the simplified model, we completely ignored the initial stress effect, and all scissor points are simplified by fixed joints connecting crossing beams. In addition, no relative rotations between beams and hubs are allowed. These simplifications are validated by the comparisons of the linear and nonlinear responses of both comprehensive and simplified models. Nevertheless, we must be aware that the assumptions also make the simulation model more stable than the real structure which provide an upper bound for the estimate of the collapse load.

**Figure 7:**
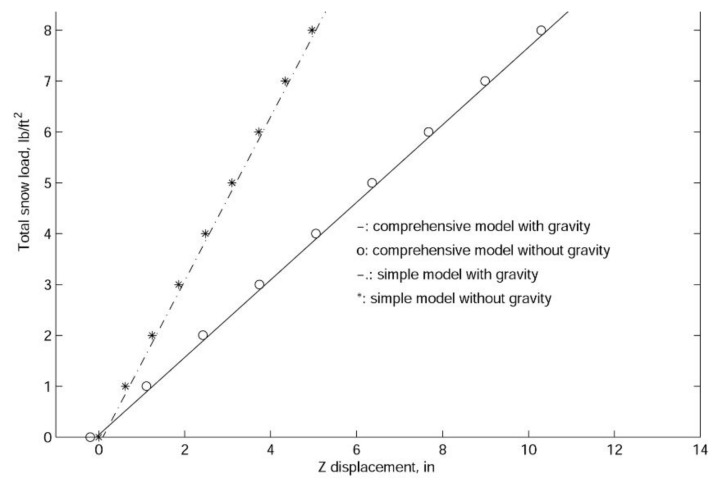
The linear load-displacement curve for the J shelter with both comprehensive and simplified models.

According to Figure 7, the linear responses of the two sets of models are very different. However, in both models, gravitational load (shelter self-weight) can be ignored. It is interesting to note that for the comprehensive model, due to the assemblage stress, the initial shelter displacement is in the opposite direction of the gravitational force, and just as for the simplified model, the gravity will slightly increase the downward displacements. It is also shown in Figure 6 that the comprehensive model is much more flexible than the simplified model, which confirms the fact that the simplification of scissor points and hubs will in fact make the model stronger than the actual structure.

To properly account for the snow load, we use the following conventional mass lumping procedure in finite element methods to redistribute the equivalent snow load to beam joints,

(9) $$F=\int_{s}H^{T}f^{s}dl,$$

where H, $f^{s}$, and F stand for the interpolation matrix, the distributed snow load, and the lumped nodal force vector, respectively.

**Figure 8:**
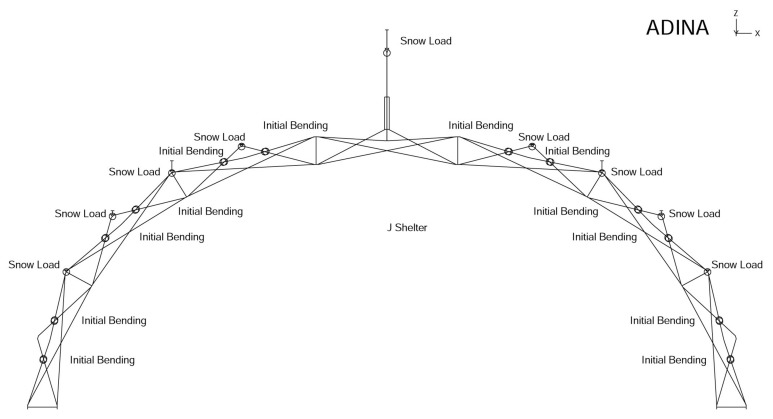
Distributed snow loads on the J shelter hubs.

The distributed snow loads are illustrated in Figure 8. At this point, we must mention the unique design of the riser, i.e., the vertical pillar. Although the riser is free to move up and down, the supports at its ends can effectively increase the column buckling load and make the riser structure more stable. Notice however, to avoid the complication of the additional buckling problem of the risers when we lump the total snow load at the center, we can gradually distribute snow load from the center of the J shelter at the top of the riser to the bottom or interior hub.

### 3.4. Design Variations

**Figure 9:**
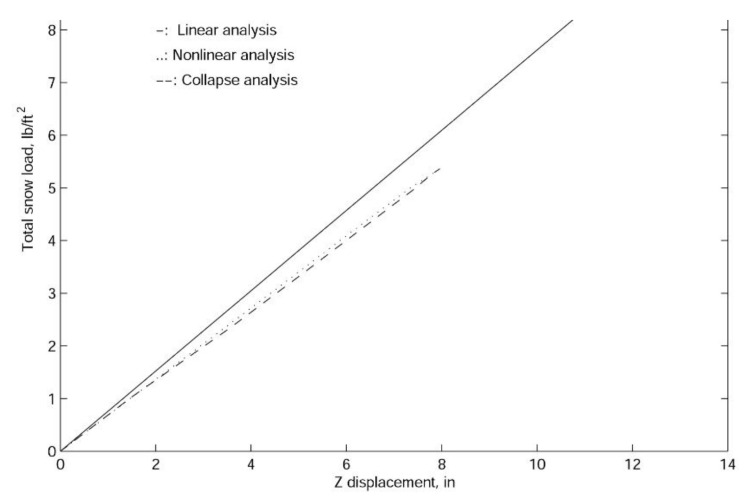
The linear and nonlinear load-displacement curves for the comprehensive model of the J shelter.

**Figure 10:**
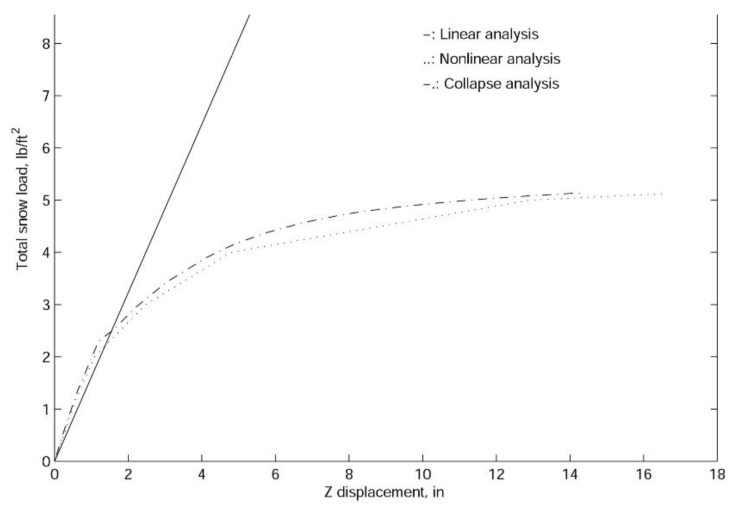
The linear and nonlinear load-displacement curve for the simplified model of the J shelter without end walls.

**Figure 11:**
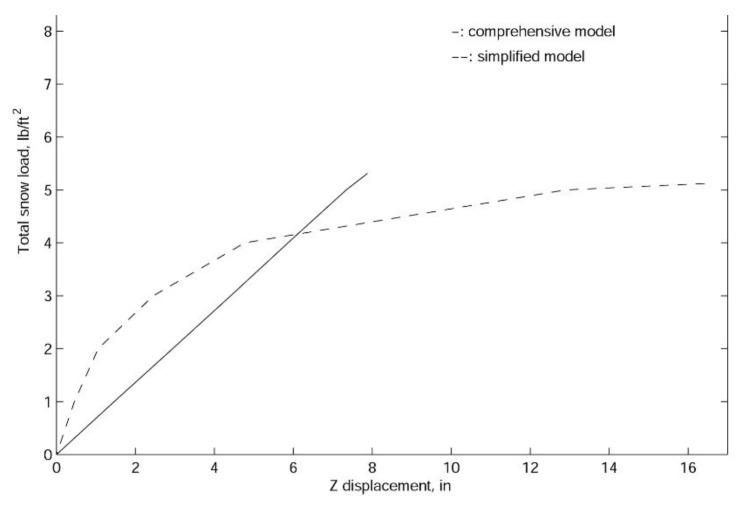
The nonlinear load-displacement curve for the J shelter with both comprehensive and simplified models.

**Figure 12:**
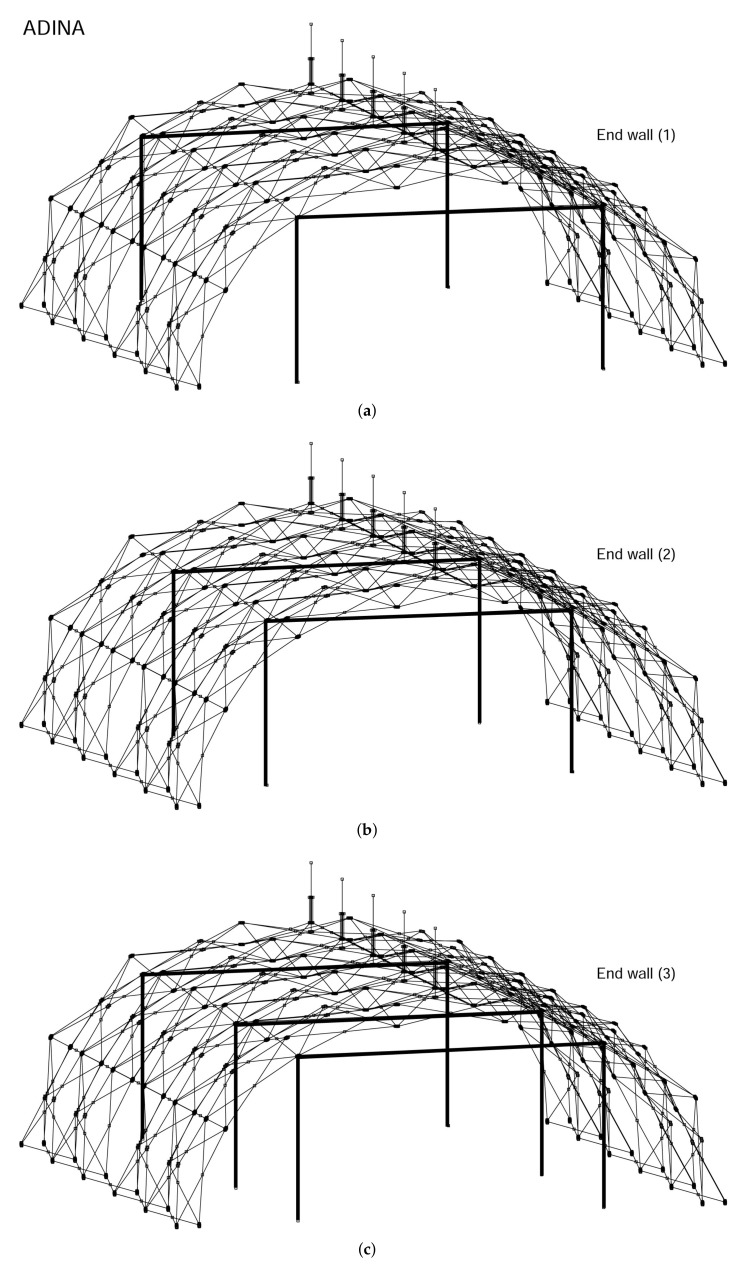
The entire structural model of the J shelter with three end wall arrangements. Notice the number of main frames represented by heavy lines and their spacing. (**a**) End Wall (1); (**b**) End Wall (2); (**c**) End Wall (3).

**Figure 13:**
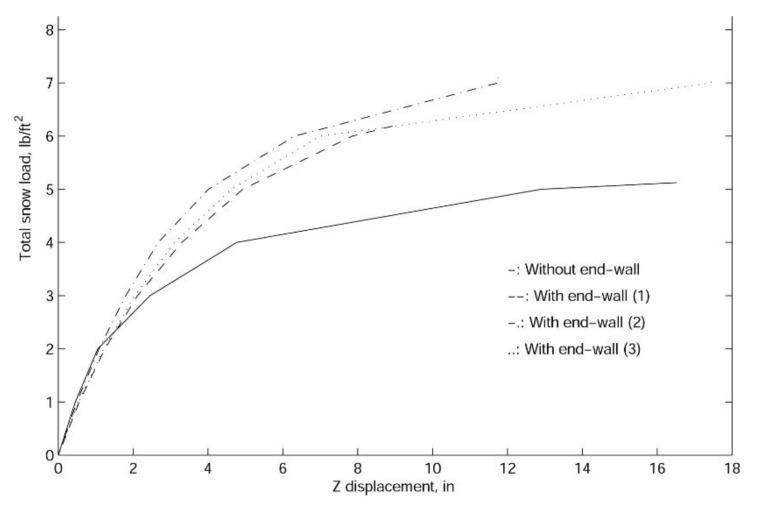
The nonlinear load-displacement curves for the simplified model of the J shelter with three different end wall locations.

In finite element analyses, the collapse load of a structure can be obtained with linearized buckling, collapse, and nonlinear analyses. In general, linearized buckling analysis is not as accurate as the other two approaches and depends very much on the starting point. However, to obtain an accurate estimation of the collapse load, in this work, we employ all three procedures. As shown in Figure 9, Figure 10 and Figure 11, both the comprehensive and simplified models predict the snow load at collapse around $5\;{\mathrm{lbf/ft}}^{2}$, which strongly suggests that the current design of the J shelter will withstand up to $5\;{\mathrm{lbf/ft}}^{2}$ snow load. Notice that although these curves in Figure 9 from nonlinear analysis are merely slightly nonlinear or curved, unlike those from Figure 10 and Figure 11, the magnitudes are significantly lower than those from the linear analysis. To explore the possible way of improvement, we also introduce a few end wall supports as illustrated in Figure 12. It is suggested from the results in Figure 13 that the second and third cases of the end wall support provide the most significant improvement of the structure stability, and the resultant snow load at collapse is increased to $7\;{\mathrm{lbf/ft}}^{2}$, a 40%. increment.

Since the entire shelter is supported by two rows of joints on each side, it is also understood from the physically intuition that the interior and exterior rows of pin points can be subjected to both compression and tension. Such distributions of reaction forces are confirmed in Table 3. Moreover, the reaction forces in Table 3 also indicate that the shelter collapses in an asymmetric mode.

## 4. Conclusions

The main contribution of this work is to establish a finite element analysis protocol for a series of DRASH shelters. In particular, an in-depth study of the J shelter is carried out using the ADINA finite element package. The key contributions of this study are two-fold: firstly, from a composite shell modeling of a typical strut, we derive the equivalent Young’s modulus and bending rigidity of the shelter strut; and secondly, we establish two structural models, one with fairly realistic finite element models for the scissor points and hubs, and the other with simplified boundary and constraint conditions. The final conclusion based on both structural models is that the maximum snow load that the J shelter can withstand is near $5\;{\mathrm{lbf/ft}}^{2}$. The possible reinforcement of the current shelter design may be accomplished by introducing end walls or improving the rigidity of the center joint of the shelter to avoid the asymmetrical buckling mode of the entire structure as well as strengthening individual struts.

In summary, through the finite element analysis and some comparison with the existing experimental data, we derive the following design information:

(1) The static load-displacement curve suggests that the structure is strong and the self-weight of the shelter can be ignored. In addition, the shelter roof displacement is relatively small under prescribed snow loads.
(2) The entire structure will collapse if subjected to $5\;{\mathrm{lb/ft}}^{2}$ snow load, which suggests that preventive procedures or additional structural reinforcements, such as end walls, must be introduced in the field before the snow accumulation reaches more than $5\;{\mathrm{lbf/ft}}^{2}$.
(3) The designs of scissor points and hubs are detrimental to the entire structural stability. In particular, hubs at certain locations are prone to rotation, and preventive measures, such as to align the hub against the rotational direction or make the hub as symmetric as possible, should be considered.
(4) The stability of risers and the rigidity of center joints are also important design factors with respect to the entire structural safety. In particular, unsymmetric collapse modes must be avoided by improving the resistance of rotations at the center joints. In particular, by introducing end walls, the resultant collapse load is increased to $7\;{\mathrm{lbf/ft}}^{2}$, a 40%. increment.

The mathematical models and finite element solutions presented in this paper clearly demonstrate the potential of using similar simulation procedures to achieve better and more optimal designs of various portable shelters with composites.

## Figures and Tables

**Table 1:** Orthotropic strut cross-sectional properties of the J Shelter.

Layer	Thickness (in)	Fiber Orientation
1	0.0111	$0^{\circ }$
2	0.0048	$88^{\circ }$
3	0.0163	$0^{\circ }$
4	0.0045	$-88^{\circ }$
5	0.0139	$0^{\circ }$

**Table 2:** Five different finite element models for the strut scissor point.

Joint Model	Deflection Results
Case 1	−0.0212207
Case 2	−0.0212207
Case 3	−0.0530516
Case 4	−0.0212207
Case 5	−0.0211484

**Table 3:** Reaction force distributions with RI, RE, LI, and LE representing the interior and exterior sides of the right and left supports, respectively.

#	RI (lbf)			RE (lbf)			LI (lbf)			LE (lbf)		
	X	Y	Z	X	Y	Z	X	Y	Z	X	Y	Z
1	−162	76	−743	437	645	1285	−64	−312	−472	−180	354	528
2	−156	143	−1550	662	38	2033	43	−197	−64	−462	−143	895
3	−115	24	−833	471	−617	1304	−64	244	−180	−390	−139	842
4	−121	−18	−899	464	611	1286	−63	−242	−187	−409	163	886
5	−152	−146	−1519	666	−34	2040	45	315	−105	−481	13	931
6	−167	−88	−751	441	−637	1289	−62	310	−461	−180	−361	533

## Data Availability

Not Applicable.
